# Wireless Technologies for Implantable Devices

**DOI:** 10.3390/s20164604

**Published:** 2020-08-16

**Authors:** Bradley D. Nelson, Salil Sidharthan Karipott, Yvonne Wang, Keat Ghee Ong

**Affiliations:** 1 Phil and Penny Knight Campus for Accelerating Scientific Impact, University of Oregon, Eugene, OR 97403, USA; bdnelson@uoregon.edu (B.D.N.); karipott@uoregon.edu (S.S.K.); 2 Department of Biomedical Engineering, Michigan Technological University, Houghton, MI 49931, USA; ymwang@mtu.edu

**Keywords:** implantable medical devices, implantable sensors, wireless communication, wireless power, wireless sensors

## Abstract

Wireless technologies are incorporated in implantable devices since at least the 1950s. With remote data collection and control of implantable devices, these wireless technologies help researchers and clinicians to better understand diseases and to improve medical treatments. Today, wireless technologies are still more commonly used for research, with limited applications in a number of clinical implantable devices. Recent development and standardization of wireless technologies present a good opportunity for their wider use in other types of implantable devices, which will significantly improve the outcomes of many diseases or injuries. This review briefly describes some common wireless technologies and modern advancements, as well as their strengths and suitability for use in implantable medical devices. The applications of these wireless technologies in treatments of orthopedic and cardiovascular injuries and disorders are described. This review then concludes with a discussion on the technical challenges and potential solutions of implementing wireless technologies in implantable devices.

## 1. Introduction

There are many instances when it is important, sometimes even vital, to have a clear picture of what is happening inside the human body. For example, the blood pressure in a coronary artery after stent placement [1], the operational status of the pacemaker post implantation [2], and the loading on an orthopedic implant [3] can all be used to optimize the treatment outcomes or prevent failure of the implants. Although non-invasive techniques such as ultrasound or X-ray imaging can assess some of these parameters [4,5], they do not provide sufficient temporal resolution needed to diagnose certain medical conditions. For those applications, implantable sensors may be the solution.

An implantable sensor is a type of implantable device that contains one or more sensing elements, such as strain gauges or pressure sensors, which perform localized measurements. One of the advantages of wireless implantable devices is that they do not need physical wires to interface with external technology. Compared to wired sensors, the wireless nature of implantable sensors leads to a different set of design considerations regarding communication and power. For example, wireless communication in tissue introduces unique challenges that are not present in wireless technologies outside the body. Biological tissues significantly attenuate wireless signals, especially at commonly used frequencies like 2.4 GHz. Safety is an additional concern because there is a need to limit the absorption of damaging energy into the tissue. It is common to use technologies that are designed for ex vivo use like Bluetooth, but these solutions are rarely ideal. Several alternative methods were researched for implantable devices [6,7], but they are not currently widely used.

Wireless devices can be broadly divided into several categories: passive (operated by an external interrogator), self-powered (with an internal battery), remotely powered (e.g., via inductive powering), or a combination of self-power and remote power (with a rechargeable internal battery). Early implantable sensors often relied on a wired connection to an output device to display the sensor readings [8]. However, most modern implantable sensors are based on various forms of wireless communication to transmit measurements to external output devices for further data processing and analysis. These wireless sensors are applied in various medical fields for diagnosis and treatments [9,10,11]. For example, implantable strain sensors are incorporated into orthopedic prosthetics to characterize the forces acting on those joints for designing better prosthetics [12], implantable cardiovascular flow and pressure sensors are used in patients with elevated risk to provide early warning of excessive clotting or impeded flow [13], and implantable neurostimulators also exist to treat muscular and neurological damage [14].

This review introduces some common wireless and sensor technologies that are used in modern implantable sensors, as well as comments on their strengths and weaknesses. Common applications in the orthopedics and cardiovascular fields are considered. Additional detailed consideration is given to wireless technologies that are used, including communication and power methods.

## 2. Wireless Sensor Technologies

**Figure 1:**
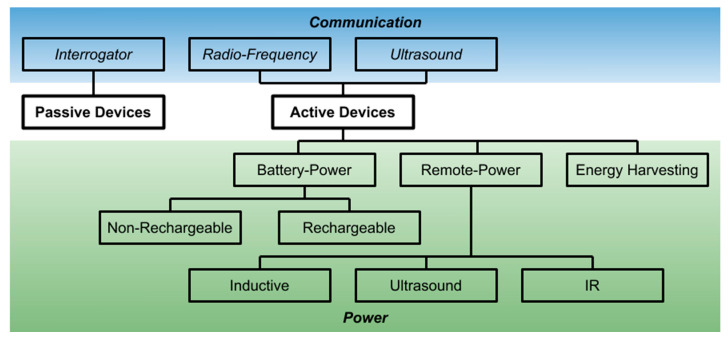
Communication and power schemes typically used in active and passive sensors.

This section provides a brief discussion of various types of wireless sensors. Although these different sensor technologies are used for various implantable devices (see Section 3), there is no one sensor or technology that is superior to the rest in all applications. In fact, the determining factor for which technology to use depends on application specifications such as the footprint/size limitation, duration of implantation, cost, safety factor, patient’s mobility requirement, etc. In this section, two types of wireless sensors (active wireless sensors and passive wireless sensors) are discussed, as each type has different limitations in sensor size, lifetime, safety, cost, convenience for data gathering, etc. In addition, two major design factors, namely, the communication and power schemes, are discussed since they also play a determining role in the sensor specification. A breakdown of different wireless sensor technologies is illustrated in Figure 1.

Passive sensors feature no active components like transistors or processors and, therefore, do not need an internal power supply for operation. These sensors have limited functions, often designed for measuring one or, at most, a few parameters intermittently over a long period. Common examples of passive sensors include inductor–capacitor (LC) or chip-less radiofrequency identification (RFID) sensors [15], which are activated with an electromagnetic field and emit a secondary field that is measured remotely, and magnetoelastic sensors, which undergo mechanical resonance when exposed to magnetic fields [16]. Passive sensors have many advantages over active sensors such as their simple design, robustness, and ability to operate for a long period of time without loss of power. However, they have limited temporal resolution and poor performance when measuring multiple or complex parameters, limiting their use as implantable sensors in many medical applications.

Active wireless sensors are sensors that contain active components such as microcontrollers and battery-powered transducers. They allow for more customization and have better performance than passive sensors, and they may include arrays of different transducers to monitor multiple analytes simultaneously. They are typically more complex and feature several major components including one or more transducers, a transceiver, a controller, and a power supply.

### 2.1. Wireless Communication

Although wireless sensors can store data locally (e.g., in flash memory) to be retrieved after some period of time, this is impractical for many applications due to the need for real-time or near real-time data collection. Furthermore, for implantable devices, directly accessing the devices to retrieve local data is highly undesirable due to the need for additional surgical procedures that reduce the safety of patients and increase cost. A simple way to collect implantable sensor data is via transcutaneous wires, but such implementation is undesirable due to increased risk of infection and movement restriction. Therefore, wired implantable sensors are usually used in short-term measurements or under controlled laboratory environments. For most clinical practice, wireless connectivity is preferred for implantable devices.

There are a broad range of wireless technologies and protocols to match the wide array of medical applications. Table 1 introduces several prominent wireless protocols (active and passive) and compares them in terms of key technical differentiators. Instead of a single wireless communication standard for all implantable sensors, governmental regulations and existing industrial standards help shape design choices for implantable sensor telemetry. Standards for communication may be found in the Institute of Electrical and Electronics Engineers (IEEE) 802.11 [17] for local area networks and IEEE 802.15 [18] for personal area networks. Many researchers and sensor developers use existing communication technologies for implantable sensors such as WiFi, Bluetooth, and ZigBee [19,20]. WiFi sensors only operate when connected to an existing WiFi network, but they can easily be connected to the internet or other devices within the WiFi network. This can be convenient in the context of locations with already existing WiFi networks, but it can introduce significant data overhead. Thread [21] is another option for designers seeking the benefits of IPv6 (Internet Protocol version 6) found in WiFi without the same levels of overhead. Bluetooth is the technology of choice for a device that is designed to connect with a device like a mobile phone or computer, and pairing to these devices is straightforward, while BLE (Bluetooth Low Energy) builds on that foundation with power-saving innovations for wireless sensors. Zigbee is associated with mesh networks containing many devices that can connect to any other device in the network, which may be convenient for a system containing several devices on the body. Note that many other proprietary and non-proprietary protocols exist in addition to these. However, due to the wide availability and low cost of these standards, the medical industry and research community are moving toward standardized protocols that are more acceptable to the general public.

#### 2.1.1. Frequency Considerations

Wireless communication for implantable sensors presents a unique set of challenges due to the electromagnetic and safety considerations that are specific to the human body. The body contains a wide variety of tissues and organs that attenuate and scatter RF signals [22]. Generally speaking, this attenuation can be minimized by using frequencies below 4 MHz, while frequencies above 1 GHz experience significantly increased scattering [23]. However, since lower carrier frequencies require larger antennae, some sensors utilize higher frequencies to minimize the overall device footprint [24].

Safety factors are important when selecting the frequency band. The Federal Communications Commission (FCC) defines two categories of electromagnetic radiation: ionizing and non-ionizing [25]. Ionizing radiation, which causes changes to biological tissue on the molecular level, is caused by radiation at frequencies in and above the ultraviolet range. Since wireless radiofrequency communications are typically below or at microwave frequencies, safety considerations for implantable wireless sensors are mainly concerned with the amount of thermal energy that is absorbed by tissue. This concern is usually addressed by limiting the power flux density and power absorption through the human body [26]. The regulations relating to what radio frequencies are available for use in implantable medical sensors vary from country to country. For example, the FCC-provisioned services like the Medical Device Radiocommunication Service (MedRadio) and the Wireless Medical Telemetry Service (WMTS) in the United States [27] contain several frequency bands for different medical devices. The International Telecommunications Union also provides several industrial, scientific, and medical (ISM) frequency bands worldwide for local communication [28].

#### 2.1.2. Security and Safety Considerations

The method for sensor communication is critical for the patient’s safety and security. Bidirectional communication provides greatly improved utility and functionality of implantable sensors. With two-way communication, the implantable sensor can employ intermittent data logging and ad hoc configuration through an external device to reduce overall power consumption since data collection and transmission are only performed on an as-needed basis. The bidirectional communication also allows firmware update of the implantable sensors post implantation, further improving the long-term reliability and safety of the sensors. Therefore, digital communication protocols such as Bluetooth or Zigbee using active sensors (see Section 2.1.1) instead of interrogation protocol with passive sensors (Section 2.2.1) would be preferred if security is a priority for the application. However, this also introduces potential security concerns [29]. For example, an unauthorized person or device can make changes to the implanted sensors and alter their functionality. Patient privacy may be compromised if unauthorized personnel are able to access and read sensor output.

Wireless transmission through human body also needs to maintain a safe level of electromagnetic energy to prevent tissue heating and meet safety standards. The specific absorption rate (SAR) as defined by IEEE is probably the most widely used method to demonstrate the safety of new wireless devices. The SAR may be calculated using the following formula:

*SAR* = (*σ*|*E*|^2^)/*ρ*, (1)

where *σ* is the electrical conductivity of the tissue, *E* is the strength of the electric field, and *ρ* is the density of the tissue. Based on this formula, it is important to design with the strength of the electric field in mind to minimize the risk to the patient. Designers must design systems with a large enough field to power the implant, but a small enough field to minimize electromagnetic energy absorption. There are multiple ways to optimize this. For example, exposure reference levels (ERLs) are higher for many lower-frequency waves due to decreased energy absorption in tissue [30]; thus, sub-GHz frequencies may be used to decrease absorption. Additionally, careful tuning of the transmitter and receiver coil can maximize received power at relatively low electromagnetic field strengths [31].

Another method used to assess the safety of wireless transmission uses the temperature of the tissue as a metric for safe exposure limits. This is done by calculating the cumulative equivalent minutes at 43 °C (CEM43°C) using the following equation:

CEM43°C = *tR*^(*T*−43)^, (2)

where *t* is the time that is considered safe for the tissue to remain at temperature *T*, where *R* is 0.5 for temperatures above 43 °C and 0.25 for temperatures below 43 °C [32]. The calculated CEM43°C may be compared with known limits for different tissues to assess how long the wireless charging is safe. This method is commonly used in ultrasound systems [33] or other systems where it is difficult or impossible to determine the SAR.

### 2.2. Power

Because active sensors require power to remain operational, the power supply is one of the most important design considerations for the long-term operation of an implantable wireless sensor. Passive sensors do not have a local power supply, but instead respond to a remote interrogator. Three methods are commonly used to power an active implantable sensor: (1) the sensor may contain a local power supply such as a battery; (2) the sensor may be remotely powered, typically with electromagnetic energy; (3) the sensor may autonomously harvest energy from its environment. The choice of power mechanism often depends on many factors including the sensor’s power consumption rate, operational period, size constraints, and safety considerations.

It should be noted that wireless communication often is the largest consumer of power in active devices. For example, a study by Mathuna et al. [34] investigating the power consumed by an environmental sensor using the Zigbee protocol found that wireless transmission and reception consumed 83 and 91 mW, respectively, compared to the 19 to 59 mW consumed by the signal processing for the various onboard sensors. These numbers can vary widely depending on many factors, such as the microcontroller, antenna design, signal processing, and voltage levels. Wireless communication is managed by the onboard processor, and designers can seek to reduce the power consumed by wireless communication by reducing the processing overhead of the microcontroller. Since most wireless sensors do not require high processor performance (complex signal processing may be performed at the receiving device), one key way to minimize power is by implementing ultra-low-power devices such as the Texas Instruments MSP430 or ARM M0+. Other ways to reduce communication overhead is by careful consideration of the communication protocol in order to balance reliable data transmission and redundant communication. For instance, popular protocols such as Bluetooth implement secondary transmissions to maintain a linked connection between the implant and external devices, and designers must consider if these secondary transmissions are in the best interest of the limited power available in implantable devices.

A passive design has the benefits of simplicity and small size and does not suffer from the limitations of budgeted power. These devices feature no active components (e.g., microcontrollers, antenna, amplifiers). Because they do not rely on battery or remote power, there is decreased risk of sensor failure over time. This allows for almost unlimited recording time, which can be useful in applications where lengthy data collection windows are desirable. However, passive sensors are often more limited in their sampling frequency than active sensors. Many passive interrogators can take up to a minute to perform a frequency sweep with high accuracy. This is impractical for applications like those in real-time orthopedic measurements where many measurements per second are necessary to identify the range of forces during motions like ambulation [35].

#### 2.2.1. Passive Interrogators

**Figure 2:**
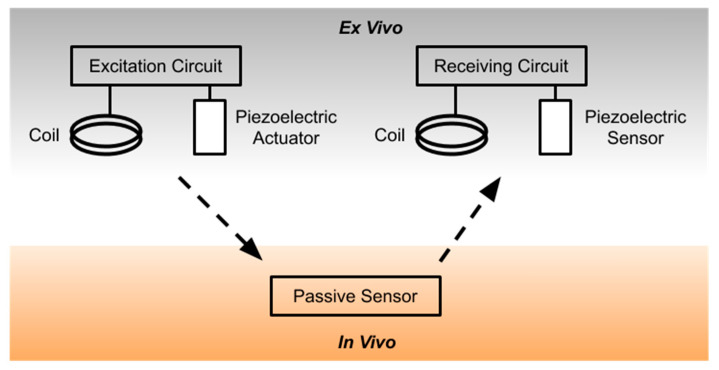
The design of a generic passive interrogator. A signal is generated in the excitation circuit and transmitted at radiofrequency (RF) using an inductive coil or at ultrasound frequencies using a piezoelectric element. The reflected signal is received by a receiving circuit using a matching coil or piezoelectric element.

Passive sensors by definition do not require local power. Passive sensors are typically resonance-based sensors that respond a known stimulus based on their mechanical properties, which can be influenced by the environment. When stimulated by a remote interrogator unit (Figure 2), passive sensors respond based on their environment and electromechanical properties to produce a signal that is measured remotely.

While there are a number of ways to passively interrogate sensors from a distance, the most common methods that are practical for implantable devices are based on electromagnetic (EM) fields (typically in radiofrequency range) [36], magnetic energy [37], and acoustic energy [38]. Electromagnetic-based passive sensors are typically made of a simple electrical resonance circuit consisting of an electrical inductor (L), capacitor (C), and resistor (R). When exposed to the EM fields, these sensors undergo resonance when it emits the highest returned signal. The parameters are typically tracked by measuring the change in the resonance frequency. In addition to the basic resonance circuit sensors that use the RLC components [36], there are a few different variations of EM-based passive sensors, such as RFID sensors [39], which have microchips that can perform more complex sensing functions, a varactor-based LC sensor that can convert voltage change to resonance frequency shifts [40], and LC sensor arrays that can measure multiple parameters simultaneously [41].

Ultrasound energy provides another means to remotely power implantable sensors. Typically, ultrasound-powered implants have energy-converting components, such as capacitive-charging units, which can generate voltages when actuated by ultrasound vibrations. To power these implants, a handheld device is used to focus the ultrasound wave onto the implant, and the measurements are transmitted back to the device using either radiofrequency or ultrasound. Such as device was implemented for implantable aorta aneurysm sensors [42]. As described in Section 2.1.1, the use of ultrasound as opposed to higher RF signals can be used to power deeper implants with less signal attenuation through tissue.

Similar to EM-based sensors, magnetic-based sensors use a magnetic field to provide the energy needed for internal measurement functions and wireless communication. Magnetoelastic sensors are a popular type of magnetic sensor made of magnetostrictive materials that exhibit physical vibrations when exposed to a magnetic field at the resonance frequency [43]. The sensors typically operate by designing or functionalizing it so that the parameters of interest alter the resonance frequency or amplitude [44]. Other magnetic sensors are based on the induction of magnetic materials, such as magnetostrictive sensors [45] or magneto-harmonic sensors [46], which change their induced magnetic field depending on the parameters of interest.

#### 2.2.2. Batteries in Implants

Many implantable devices contain local power supplies such as batteries or supercapacitors. The incorporation of a battery into an implantable device can significantly increase the size of the implant. In many cases, this is undesirable. However, batteries allow the device to be used anywhere so that the user does not have to be in the presence of a transmitter. This makes them extremely useful in implantable devices such as pacemakers and defibrillators that need to function 24 h a day [47]. Batteries are capable of providing relatively consistent power throughout the lifetime of the device without the need for external equipment. However, non-rechargeable batteries have a limited lifetime before they must be replaced or discarded. Roughly 25% of pacemaker-related surgical interventions are due to battery replacement [48]. This leads to the limitation of sensor lifetime for a given battery size. Long-lasting batteries, typically used in pacemakers, are much too large for many implantable applications; thus, smaller batteries with shorter lifetimes must often be used. As a result, battery-powered designs are most appropriate for larger devices (such as pacemakers and defibrillators). Some devices implement secondary (or rechargeable) batteries to circumvent this limitation. Rechargeable implants may be recharged using any methods for remote power or energy harvesting.

#### 2.2.3. Remote Power

Unlike true passive sensors, many battery-free sensors have active internal electronic circuitry that still need power to operate by obtaining the power remotely. These battery-free sensors are in use since decades ago (e.g., Reference [49]). These remote power schemes provide an appropriate alternative to battery power for sensors that operate intermittently or a method to recharge onboard batteries. They do not suffer from the limited lifetime of traditional battery-powered sensors, and they can offer greater flexibility in terms of lifetime than other devices of comparable size. However, they can be limited by the need to be in proximity to a power source or recharging station.

**Figure 3:**
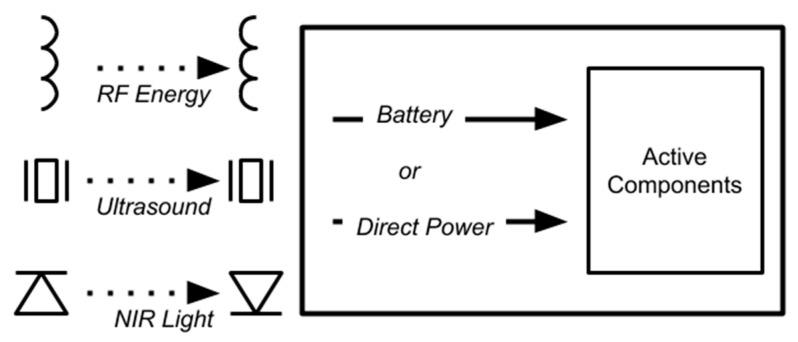
Methods of remote power for implantable devices. RF energy (top-left) can be transmitted through inductors; ultrasound energy (middle-left) can be transmitted through piezoelectric materials; near-infrared (NIR) light (bottom-left) can be transmitted via light-emitting diodes (LEDs) and photoreceptors. The received energy may be either stored in a battery or used directly.

There are several common methods (Table 2, Figure 3) for transmitting power through tissue to an implantable device. Radiofrequency or inductive power transfer has the potential to be used in devices like pacemakers and defibrillators because it can prolong the lifetime of the battery [50]. This method uses a coil antenna as a transmitter, which generates electromagnetic waves around the coil. A second coil antenna acts as a receiver and converts the electromagnetic energy into electrical current. The directionality of the electromagnetic field can be improved using ferrite shielding. Inductive power can produce increased heat at the implant site, which may be a safety concern [51]. One of the greatest challenges in inductive power transfer is the loss of energy through tissue, which can be reduced at sub-GHz frequencies [52]. Recent developments like in vivo networking (IVN) are seeking to overcome this limitation by implementing multiple transmitters to provide power and communication to deep implants [7].

Ultrasound or acoustic power transfer is another commonly used method [56,58,59,60,61] because it features significantly higher efficiency than inductive methods at greater distances between transmitter and receiver [62], and because of its more precise directionality resulting in increased power transfer efficiency and decreased tissue damage [63]. Ultrasonic power is commonly implemented in mm-sized implants because the size of the receiver can be much smaller than an inductive coil. They use piezoelectric transmitters and receivers to transmit at frequencies of 3–6 MHz [62].

Some researchers proposed the use of infrared (IR) light sensors for remote power or recharging of implantable devices. This method uses superficial photodetectors to receive near-infrared light, which passes through the skin with relatively high efficiency [64]. The use of IR light as an energy source is more commonly applied to collect ambient light rather than for directed energy transfer because the receiver must be very superficial, a limitation that is not present in inductive or ultrasound energy transfer.

For all methods of remote power, accurate relative alignment of the transmitter and receiver is critical. Both the absolute distance and the lateral distance (i.e., misalignment) between the transceivers have an extremely significant impact on power transfer efficiency. However, different methods of wireless power transfer have different relationships. Simulations show that the intensity of transmitted ultrasonic power remains relatively consistent as absolute distance from the transmitter increases [53], and omnidirectional transceivers were proposed to minimize loss of intensity with misalignment [65]. Conversely, loosely coupled electromagnetic power transfer decreases sharply as absolute distance from the transmitter increases, but it is less susceptible of misalignment [66]. Both tightly coupled electromagnetic and light power transfer tend to require more precise positioning in both planes.

#### 2.2.4. Energy Harvesting

Many medical device applications need constant or near constant power, but with a limited footprint and the need for a longer lifetime than a typical battery can provide. It is often undesirable to replace the battery in long-lasting devices due to the inconvenience, cost, and risks associated with battery-replacement surgery. To accomplish these objectives, many researchers found ways to harvest energy from the environment of the implant for an extended period of time. Some examples of this include harvesting solar energy or energy from the motion of the body. Other methods such as harvesting thermal energy [67] were proposed, but they show limited promise for in vivo applications. These sensors contain active electrical components similar to remote-powered sensors; however, instead of relying on external power source, they obtain power from their environment.

One promising method for energy harvesting is the use of photovoltaic power (Table 3). Near-infrared (NIR) light present in sunlight can pass through skin and tissue with low power losses [71]. Various subcutaneous solar panels were proposed for biomedical implants [55,72] and implemented in pacemaker designs [68,69]. Studies suggested that the power harvested by these solar panels is sufficient to power a pacemaker [73], as well as devices that use wireless communication [70], even in cloudy weather or winter months. However, their efficacy may vary based on skin tone or things covering the implant site. This method lacks the added benefit of being able to transmit both power and data over the same energy spectrum.

There are a variety of transducers available to convert kinetic energy into electrical energy (Table 4). A piezoelectric material, which produces a voltage when it is mechanically strained, may be most appropriate in situations where large strains are present, such as in orthopedic and cardiovascular applications. Piezoelectric materials generate a net charge displacement when the crystal structure is augmented through strain. This charge displacement can be converted and stored for use in electronic devices. There are many wearable piezoelectric energy harvesters that use forces that occur during ambulation [77], and some implants use a similar approach at joint interfaces [74]. Other implantable devices using piezoelectric energy harvesting typically rely on the motion of the heart or lungs. Examples of this technology include harvesting energy from diaphragm motion [78] or from the displacement of the aorta due to circulation [79]. Electrostatic or triboelectric energy harvesters are also used to convert motion, such as respiratory motion [75], into energy.

## 3. Wireless Medical Sensors

Although many non-implantable diagnostic techniques and imaging technologies are available for disease detection and monitoring such as magnetic resonance imaging [80], diagnostic ultrasound [81], and X-rays [82], there are many clinical applications in which implantable sensors are more effective, safe, and/or convenient to use. Therefore, wireless sensor technologies, such as those described in Section 2, can be incorporated into existing implantable devices or used as standalone diagnosing devices. This section summarizes some common medically relevant parameters in orthopedics and cardiovascular applications in which implantable sensors are appropriate.

### 3.1. Orthopedics

Diagnostic techniques are commonly required to assess orthopedic injuries. Soft-tissue orthopedic injuries represent about 35% of lost work days each year in the United States, accounting for more than $20 million a year in worker compensation. There are an estimated two million bone fractures in the United States annually, many of which are linked to bone diseases [83]. Severe bone fractures often require invasive fixation devices (either internal or external) for proper healing. Furthermore, joint loosening, which may be caused by arthritis, is often treated with prosthetic implants [84]. Implantable sensors such as those in Table 5 are applied along with prosthetics or fixation devices to monitor fracture healing and/or acquire motion feedback.

#### 3.1.1. Smart Prosthetics

**Figure 4:**
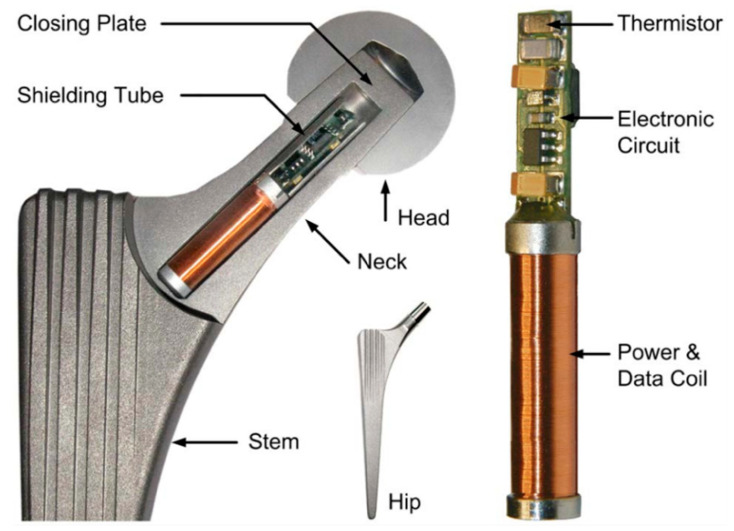
A smart hip implant featuring a telemetered temperature sensor to detect loosening. CC BY [85].

One major application of implantable wireless sensors is to allow sensing capability in a prosthetic implant to create a smart prosthesis, as shown in Figure 4. One early smart prosthetic hip used an array of 14 pressure transducers arranged inside the femoral head to characterize the distribution of forces within the joint during motion [88]. Other sensors incorporated into hip implants used strain gauges [89,90] or thermistors [85] as transducers. Similar devices were implemented in femoral implants [86,91], shoulder implants [92], and both vertebral [93] and interbody [94] spinal implants.

Sensors are also implemented to directly monitor body conditions without the addition of a fixation device or prosthesis. This sensor design is of particular interest in preventative care, as well as in biomechanics research, where it is undesirable to alter the structural mechanics of the bone. Sensors of this nature are implemented to collect both strain and force data. The strain sensor, developed by Szivek et al. [95], is used to detect spinal fusion. In this design, several resistive strain gauges were bonded to the spine and wired to a subcutaneous telemetry device produced by Microstrain, Inc. (Burlington, VT, USA). A piezoresistive pressure sensor [96] was similarly implanted in pigs to monitor intradiscal hydrostatic pressure using the V-Link wireless sensor node (Microstrain, Inc., Burlington, VT) [97]. These smart prosthetics are usually active devices powered by battery or remote power.

#### 3.1.2. Fracture Healing

In clinical settings, an accurate understanding of the state of fracture healing is important to advise patients on when and how they may load the injured bone, as well as for determining when a fixation device may be safely removed while minimizing risk of re-fracture [98,99]. This is also vitally important in preclinical settings for better understanding and quantifying the effectiveness of new treatments [10]. To address this need, many implantable orthopedic sensors were developed to monitor the forces acting on internal fixation devices. These devices are commonly used to hold a group of bones in a desirable alignment (e.g., for scoliosis patients) or to hold a fractured bone in place so that it heals properly. One of the earliest implantable sensor nodes [49] was a spinal fixator that monitored the forces acting on the spine. Prior to this device and its use of wireless telemetry, such measurements could only be taken using transcutaneous wires for data collection [100]. A similar design is used in spinal fixation devices [101], as well as in implantable fixators for the hip [102,103] and femur [35,104].

Determining the size of the fracture callus, which is commonly performed with X-ray, is important because callus size and stiffness directly correspond to healing progress, and improperly healed fractures are prone to re-fracture [98]. However, standard X-ray images cannot provide a strong indication of the mechanical properties of the callus, nor can it provide continuous measurements [4]. An alternative method to determining callus stiffness indirectly is the use of a strain gauge attached to the fixation device [105,106]. In this method, the compressive load supported by the fixation device decreases as the fracture heals. This method is used in implantable devices to provide real-time measurement over the course of the healing process [35,107].

### 3.2. Cardiovascular

Implantable devices are also widely used in cardiovascular applications. Cardiovascular disease (CVD) is attributed to as many as 45% of all deaths in Europe [108]. In the United States alone, about 720,000 people suffer heart attacks every year [109]. Many of these heart conditions are caused by blockages or stenosis, narrowing the blood supply to the heart muscle. A multitude of companies are working to develop implantable cardiac sensors such as those in Table 6 to monitor heart failure and other varieties of CVD.

#### 3.2.1. Blood Pressure Monitoring

Many studies confirmed a correlation between high blood pressure and increased cardiovascular disease in people aged from 30 years to over 80 years [112]. In 2010, high blood pressure was the leading cause of death and disability-adjusted life worldwide [112]. From 2004 to 2014, the number of deaths caused by high blood pressure increased by 34.1% [109]. Monitoring blood pressure could provide insight into preventing its escalation before complications that are more difficult to treat, such as disability progression in hypertensive multiple sclerosis patients [113], sleep apnea [114], and dementia [115].

The first long-term, implantable blood pressure sensor was described by Van Citters and Franklin [116] and was used in several mammals. This design features a diaphragm-based pressure sensor which is implanted directly into the lumen of a major vessel, most commonly the aorta or first intercostal vessel. Measurements from the sensor are transmitted using frequency modulation at a center frequency of 14.5 kHz. These sensors were effectively demonstrated in vivo for more than a year. A similar device is also implanted into the ventricular wall [117].

Pulmonary arterial blood pressure monitoring has great clinical significance. Research demonstrated that heart failure can be detected up to weeks in advance by monitoring changes in blood pressure [118]. Therefore, there is a need for non-invasive technologies to monitor blood pressure. One prominent solution to this is the CardioMEMS^TM^ HF System (CardioMEMS^TM^ Inc., Atlanta, GA, USA). This solution is a passive LC sensor that is implanted into the pulmonary artery using a catheter to monitor changes in pressure. Studies showed that this sensor is able to provide relevant data to clinicians and reduce hospitalization for at-risk patients [119,120].

Intravenous catheter-style sensors are also implanted to monitor blood pressure. Brockway et al. [121] used a piezoresistive catheter inserted into the aorta of a rat to monitor ambulatory heart rate and blood pressure. This telemetry system later became available via Data Science International [122]. Catheter-based pressure sensors are also used to monitor bladder pressure in pigs [123].

**Figure 5:**
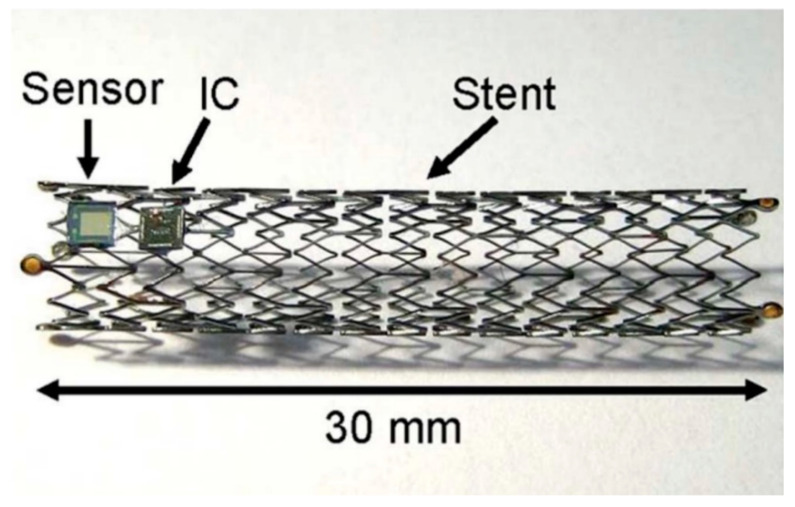
An example of a remote-powered blood pressure sensor. Power is received via the stent, which doubles as an antenna, to power the sensor and processing integrated circuit (IC). Reprinted with permission from Reference [110].

Extravenous cuffs are used to provide sensing that is less invasive to the circulatory system. Cong et al. [124] demonstrated a cuff featuring a capacitive pressure sensor implanted around the carotid artery in rodents. Similar cuff designs are also incorporated into pulmonary stents as shown in Figure 5 [110,125]. These cuffs, which are powered via inductive coupling, use the stents as antennas for communication. Tonometric cuffs were also demonstrated [126,127]; these devices slightly constrict the blood vessel with a known pressure, while an array of capacitive pressure sensors relay pressure measurements to the telemetry device.

#### 3.2.2. Blood Flow Monitoring

Blood flow monitoring is essential in studying and treating a vast array of detrimental health problems and, in particular, neurodegenerative disorders. Cerebral blood flow regulation is necessary for normal brain function and upon interruption, and it can cause irreversible damage to neurons. Without regular cerebral blood flow, impairment of brain functions can occur, resulting in neurovascular dysfunction and a multitude of conditions including Alzheimer’s disease [128]. In addition, blood flow monitoring can help prevent lower-limb amputations in diabetic patients with peripheral occlusive arterial disease [129] and can facilitate the assessment of myocardial viability [130]. Many implantable blood flow sensors also use the extravenous cuff design featuring Doppler sensors [111,131]. Electromagnetic flow sensors are similarly attached to the outside of vessels for blood flow monitoring [132].

## 4. Conclusions

Wireless implantable sensors are seeing increased popularity for various medical applications. While currently there are certain practical issues for many implantable sensors, rapid improvements in battery technologies, communications protocols, and sensor fabrication techniques will overcome these challenges and allow wider adoption of these sensors to improve healthcare. For example, although the performance period of active sensors is always limited by the power supply, careful consideration of the power schemes, such as invoking low-power mode or using wireless charging, can significantly reduce or even eliminate this shortcoming.

Today, wireless implantable sensors are used for monitoring orthopedic implants, cardiovascular environments, and more. Although most of these sensors are used for research purposes, some are clinically implemented such as the Verasense^TM^ for quantifying alignment of knee arthroplasty and CardioMEMS^TM^ sensors for blood pressure monitoring. With better and wider acceptance of implantable technologies, it is expected that more of these sensors will be successfully developed for clinical uses in the near future, further improving the diagnosis of many diseases and monitoring the outcomes of medical treatments.

Power and communication remain major limitations in implantable devices. The wide implementation of Bluetooth in many technologies that we interact with every day is likely due to the robust standardization of the communication protocol, allowing it to be used in many types of devices. The development of a standard more appropriate for implants may have a similar effect on the wider use of implantable technologies. An ultra-low-power protocol operating at sub-GHz frequency is a good candidate for such a standard. Similarly, improvements in energy harvesting or efficient and safe remote power methods will boost the reliability of implantable devices and reduce the need for additional interventions aimed at repair or replacement.

Implantable wireless sensors play an invaluable role in showing researchers and clinicians what hidden problems may be present in the body. Their major strength is their ability to quantify events happening within the body in real time in a non-invasive method. However, there is a significant regulatory burden on each device, which often limits their use to preclinical applications. There remain many areas of improvement for implantable devices, including more robust communication and decreased size. Both of these areas may be avoided using wearable sensors, and it is likely that distinct applications will emerge where the convenience of wearable technology supersedes the benefits of implantable technology and vice versa.

## Figures and Tables

**Table 1:** Comparison of common communication protocols. BLE—Bluetooth Low Energy; IPv6—Internet Protocol version 6; N/A—not applicable.

	BLE	Zigbee	Thread	WiFi	Passive
Frequency	2.4 GHz	2.4 GHz	2.4 GHz	2.4 GHz	N/A
Bandwidth	1 MHz	2 MHz	2 MHz	22 MHz	N/A
Mesh Capable	Yes *	Yes	Yes	Yes	No
IPv6 Addressable	No	No	Yes	Yes	No
Encryption	AES	AES	AES	WEP/WPA	None

* Supported in version 5.0 or later.

**Table 2:** Typical metrics of ultrasound, electromagnetic, and light remote power (or wireless power transfer) methods.

	Ultrasound	Electromagnetic	Light
Frequency	200 kHz–1.2 MHz [53]	1 MHz–3 GHz [54]	220 THz–460 THz [55]
Receiver	Piezoelectric element	Antenna	Photovoltaic cell
Depth	Over 10 cm [56]	Up to 5 cm [57]	Less than 5 mm
Misalignment Resilience	Moderate	Very Low	Low

**Table 3:** Comparison of select photovoltaic energy harvesting implants.

	Haeberlin 2015 [68]	Haeberlin 2014 [69]	Wu 2018 [70]
Depth (mm)	2.4	3.1	3
Location	Neck (pig)	Abdomen (pig)	Skin flap
Average Power (mW)	6.747	15.448	0.6–5.5
Power/Area (mW/cm^2^)	1.417	4.768	0.025–0.234

**Table 4:** Comparison of select kinetic energy harvesting implants.

	Platt 2005 [74]	Zheng 2014 [75]	Dagdeviren 2014 [76]
Transducer	Piezoelectric	Triboelectric	Piezoelectric
Location	Knee (ex vivo)	Lungs (rat)	Heart (cow)
Average Power (mW)	4.8	0.0005	0.0012

**Table 5:** Comparison of select smart orthopedic implants. EM—electromagnetic.

	Bergmann 2012 [85]	D’Lima 2005 [86]	Klosterhoff 2020 [87]
Application	Implant failure detection	Force measurement	Fracture repair monitoring
Power Supply	4 kHz EM	EM	Battery
Communication	EM	EM	BLE
Sensor	Thermistor	Strain gauges	Strain gauge

**Table 6:** Comparison of select cardiovascular wireless implants.

	Chow 2009 [110]	CardioMEMS^TM^	Yeshwant 2019 [111]
Application	Blood pressure monitoring	Heart failure detection	Blood flow monitoring
Power Supply	2.4 GHz EM	Passive	Battery
Communication	2.4 GHz EM	N/A	EM
Sensor	Pressure	Pressure	Pressure
